# Frequency distribution of hepatitis C virus genotypes in different geographical regions of Pakistan and their possible routes of transmission

**DOI:** 10.1186/1471-2334-8-69

**Published:** 2008-05-23

**Authors:** Muhammad Idrees, Sheikh Riazuddin

**Affiliations:** 1 Division of Molecular Virology & Molecular Diagnostics, National Centre of Excellence in Molecular Biology, University of the Punjab, Lahore 53700, Pakistan

## Abstract

**Background:**

Information regarding hepatitis C virus genotypes and subtypes circulating in Pakistan and various risk factors for their transmission are not known well. The specific objective of this study was to find out the frequency of various HCV genotypes present in well-characterized Pakistani HCV isolates and their possible routes of transmission.

**Methods:**

A total of 3351 serum samples were tested by type-specific genotyping assay. Out of 3351 HCV RNA positive patients, 2039 were males and 1312 were females. As regard as genotyped samples, 2165 belonged to Punjab region, 823 belonged to N.W.F.P., 239 to Sindh and 124 patients were from Balochistan.

**Results:**

Out of the total 3351 tested serum samples, type-specific PCR fragments were observed in 3150 (94.00%) serum samples. The distribution of genotypes of the typeable samples as determined by this assay, was as follows: 1664 (49.05%) genotype 3a; 592 (17.66%) genotype 3b; 280 (8.35%) genotype 1a; 252 (7.52%) genotype 2a; 101 (3.01%) genotype 1b; 50 (1.49%) with genotype 4; 25 (0.75%) with 3c; 27 (0.80%) genotype 2b; 6 (0.18%) with subtype 5a; 5 (0.15%) genotype 1c; 4 (0.12%) with subtype 6a; 3 (0.09%) genotype 2c; and 161 (4.80%) patients were infected with mixed infection. Two hundred and one (5.99%) serum samples were found untypeable by the present genotyping system. More than 86% and 72% patients with genotypes 3a and 3b respectively had received multiple injections in past. For genotypes 1a and 1b the route of transmission was major/minor surgery along with unknown reasons. Majority of the cases with type 2a, 2b and indeterminate genotypes were sporadic. Mixed infections were common in thalassaemic patients.

**Conclusion:**

The most common HCV genotype in Pakistan is type 3a. Regional difference in genotypes was observed only in Balochistan province of Pakistan. More than 70% of the cases were acquired in hospitals through reuse of needles/syringes and major/minor surgery that is very common in this country.

## Background

Hepatitis C virus (HCV) infection is one of the most important *Flaviviridae *infections with significant clinical problems throughout the world in humans and it is responsible for the second most common cause of viral hepatitis [1]. To date at least six major genotypes of HCV, each having multiple subtypes, have been identified worldwide [2]. The different genotypes are relevant to epidemiology, vaccine development, and clinical management of chronic HCV infection [3]. Furthermore, the HCV genotype is the strongest predictive parameter for sustained virological response [4]. This clinical relevance of HCV genotyping attracted attention from studies that reported an influence of HCV genotypes on the clinical course of disease and response to interferon therapy, as patients with different HCV genotypes respond differently to alpha interferon [5]. Firm evidence has been established that patients with type 2 and type 3 HCV infections are more likely to have a sustained response to therapy than patients with type 1 HCV infections [6]. Rates of sustained virological response to combination therapy in patients infected with HCV-2/3 and HCV-1 genotypes are 65% and 30%, respectively [7,8]. Therefore the patient genotype should be taken into consideration when prescribing interferon standard therapy.

HCV genotypes 1, 2, and 3 appear to have a worldwide distribution and their relative prevalence varies from one geographic area to another. HCV subtypes 1a and 1b are the most common genotypes in the United States [4]. These subtypes also are predominant in Europe [9-11]. The predominant subtype reported from Japan is subtype 1b that is responsible for up to 73% of cases of HCV infection [12]. HCV subtypes 2a and 2b are relatively common in North America, Europe, and Japan and subtype 2c is found commonly in northern Italy. HCV genotype 4 appears to be prevalent in North Africa and the Middle East [13,14], and genotypes 5 and 6 seem to be confined to South Africa and Hong Kong, respectively [15,16]. HCV genotypes 7, 8, and 9 have been identified only in Vietnamese patients [17], and genotypes 10 and 11 were identified in patients from Indonesia [18]. There has been disagreement about the number of genotypes into which HCV isolates should be classified. Investigators have proposed that genotypes 7 through 11 should be regarded as variants of the same group and classified as a single genotype, type 6 [19,20].

From Pakistan few studies are available on the distribution of various hepatitis C virus genotypes based on small sample sizes [13,21,22]. No study is available on geographic variation in the prevalence of various HCV genotypes and various routes of transmission for different genotypes from Pakistan. Therefore, this study was initiated to determine the frequency distribution of various HCV genotypes and subtypes present in different geographical regions of Pakistan and to determine various risk factors for its transmission.

## Methods

### Source of Clinical Samples

All the serum samples were received along with specifically designed data sheets at Division of Molecular Virology & Molecular Diagnostics, Centre for Applied Molecular Biology, Lahore from 121 tertiary collection centers situated in different cities/towns of all the four provinces of Pakistan such as North West Frontier Province (NWFP), Punjab, Sindh and Balochistan for the detection of HCV during the course of this study. Pakistan is situated in the western part of the Indian subcontinent, with Afghanistan and Iran on the west, India on the east, and the Arabian Sea on the south. Pakistan is the sixth most populous country in the world. Pakistan is a federation of four provinces (described above), a capital territory and federally administered tribal areas. The total land area is estimated at 803,940 square kilometers. Estimated (2007) population of the country is 169,270,617. Serum samples from chronic HCV carriers showing HCV RNA positivity with all the required information representing the four provinces of Pakistan were included in the study. The N.W.F.P was represented by cities/towns of Peshawar, Mardan, Malakand, Dir, Swat, DI Khan and Bunnu (number of isolates [*n*] = 823); the Punjab was represented by Lahore, Gujranwala, Mandi Bahauddin, Multan, Sarghoda, Jehlum, Sialkot, Rawalpindi, Rahim Yar Khan and Faisalabad (*n *= 2165); the Sindh was represented by Karachi, Hydarabad, Khair Pur, Nawab Shah, Dadu and Mirpur Khas (*n *= 239); and Balochistan was represented by Quetta, Ziarat and Sibbi (*n *= 124). The isolates from NWFP, Punjab, Sindh, or Balochistan were designated as NP, PP, SP, or BP, respectively, to identify the origin of the samples. A written informed consent was taken from patients and the data sheet contained demographic of patients, possible route of transmission, area, and estimated time of infection and complete address of the patients with telephone numbers. The study was approved by the ethics committee of the Institute.

### HCV RNA Detection and quantitative PCR

Nested reverse transcriptase (RT) PCR was carried out for the qualitative detection of HCV RNA using primers corresponding to 5'NCR primers as described previously [23]. Briefly, RNA was extracted from 100 μl serum samples using Gentra (Puregene, Minneapolis, MN 55441 USA) RNA isolation kit according to the protocol described in the kit. Moloney murine leukemia virus (MMLV) reverse transcriptase enzyme (RTEs) (Invitrogen, Corp., California USA) was used for reverse transcription of HCV 5'NCR with outer antisense primer. First-round and nested PCRs were carried out with Taq polymerase (Invitrogen, Corp., California USA), and the specific HCV PCR bands were visualized on 2% agarose gel. Quantification of HCV RNA was performed for PCR positive sera with SmartCycler II Real-time PCR (Cepheid, Sunnyvale, Calif. USA). The SmartCycler II system is a PCR system by which amplification and detection are accomplished concurrently with TaqMan technology (Applied Biosystems, Foster City, Calif) using fluorescent probes to detect amplification after each replicating cycle. This assay has lower and upper detection limits of 5.0 × 10^2 ^and 5.0 × 10^7 ^IU/mL, respectively. Specimens yielding values above the upper limit were routinely diluted 100-fold and retested and obtained values were multiplied by this dilution factor to obtain the actual HCV RNA concentration in international units per mL.

### HCV Genotyping

HCV genotyping was carried out using type-specific HCV genotyping method as described previously in detail [24]. Briefly, about 50 ng of HCV RNA was reverse transcribed to cDNA using 100 U of M-MLV RTEs at 37°C for 50 minutes. Two μl of synthesized cDNA was used for PCR amplification of 470-bp region from HCV 5'NCR plus core region by first round PCR amplification. As there were 12 different HCV types that we tried to detect, so the type-specific primers were divided into two groups on the basis of differences in the sizes of the different bands, so that no genotype-specific bands are of same size in the same group on agarose gel. Therefore, two second-round PCRs were performed for each first round PCR sample, one with primer mix A and the other with mix B in a volume of 20 μl with nested PCR primers. Mix-A contained primers for genotypes 1a, 1b, 1c, 3a, 3c and 4 and Mix-B included the 2a, 2c, 3b, 5a, and 6a primers. The second round PCR product was electrophoresed on a 2% agarose gel to separate type-specific fragment. Stained the gel with ethidium bromide and evaluated under UV transilluminator. A 100-bp DNA ladder (Invitrogen, Corp., California, USA) was run in each gel as DNA size marker and the HCV genotype for each sample was determined by identifying the HCV genotype-specific PCR band.

### Sequencing of 5'NCR

The 5'UTR region of all the 201 untypable isolates were sequenced in both directions and sequences that were obtained for the first time were submitted to GenBank data base. The Accession Numbers provided for our nucleotide sequences by the GeneBank are from EF173931 to EF174030.

### Statistical analysis

The data was analyzed and the summary statistic was carried out by a statistical package, SPSS version 10.0 for window. The results for all variables were given in the form of rates (%). Chi Square and Fisher's Exact tests were used for categorical variables that measured association among categorical variables. All data are presented as mean values or number of patients. *P*-values less than 0.05 were considered significant.

## Results

### Demographic of patients

**Figure 1:**
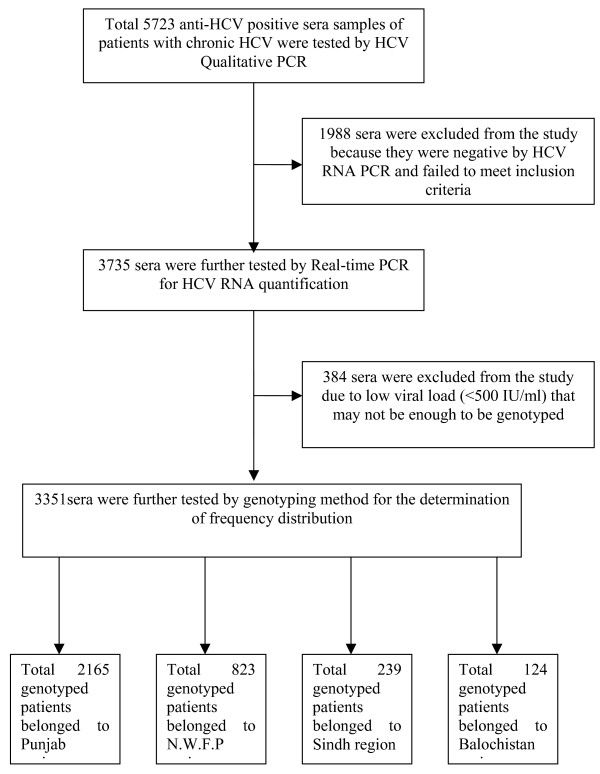
**Study enrollment of patients**. For study enrollments the patients were required to have chronic HCV with positive anti-HCV ELISA. The patients were also required to have detectable HCV RNA by qualitative RT-PCR and viral load >500 IU/ml and belonged to any of the four provinces of Pakistan

Figure 1 shows the study enrolment of patients and exclusion criteria. During the course of this study, total of 5723 anti-HCV positive sera were received from all the four provinces of the country. Out of these 3735 samples were found positive by HCV qualitative PCR. After HCV RNA quantification, 384 samples were excluded from the study due to low viral load (<500 IU/ml) and 3351 serum samples with viral load >500 IU/ml were tested by type-specific genotyping assay. Out of 3351 patients, 2039 were males and 1312 were females. Total of 2165 genotyped patients belonged to Punjab region, 823 patients to N.W.F.P., 239 to Sindh and 124 patients were from Balochistan. All serum samples tested were HCV-RNA positive with enough viral load and could thus be genotyped by genotype-specific PCR assay.

### Distribution of HCV genotypes in the study population

Table 1 shows the distribution of HCV genotypes in the study population. Out of the total 3351 tested serum samples, type-specific PCR fragments were observed in 3150 (94.00%) serum samples. The distribution of genotypes of the typeable samples as determined by this assay, was as follows: 1664 (49.05%) were genotype 3a; 592 (17.66%) genotype 3b; 280 (8.35%) genotype 1a; 252 (7.52%) genotype 2a; 101 (3.01%) genotype 1b; 50 (1.49%) genotype 4; 25 (0.75%) 3c; 27 (0.80%) genotype 2b; 6 (0.18%) subtype 5a; 5 (0.15%) genotype 1c; 4 (0.12%) subtype 6a; 3 (0.09%) genotype 2c; and 161 (4.80%) patients were infected with mixed infection. Two hundred and one (5.99%) serum samples were found untypable by the present genotyping system. Six genotypes i.e. 1c, 2c, 3c, 4, 5a and 6a were isolated for the first time from Pakistan in the course of the present study. The 5'UTR region of all the 201 untypable isolates were sequenced in both directions and sequences that were obtained for the first time were submitted to GenBank data base. The Accession Numbers provided for our nucleotide sequences by the GeneBank are from EF173931 to EF174030.

**Table 1:** Frequency distribution of HCV genotypes and subtypes in the studied population (n = 3351)

***HCV Genotype***	***HCV*^γ ^*Subtype***	***No. of Isolates***	***Percentage***
1	1a	280	8.35
	1b	101	03.01
	1c	5	0.15
2	2a	252	07.52
	2b	27	00.80
	2c	3	00.09
3	3a	1644	49.05
	3b	592	17.66
	3c	25	0.75
4	4	50	01.49
5	5a	06	00.18
6	6a	04	00.12
Mixed^α^		161	04.80
Undetermined^β^		201	05.99
Total		3351	100

^α^Mixed, two genotypes in one patients such as 3a+1a, 3a+3b etc.

^β^Undetermined, as the genotype assay was unable to assign type and these may be new subtypes.

^γ^HCV, hepatitis C virus.

### HCV genotypes in male and female patients

The distributions of HCV genotypes were similar in male and female patients.

### Frequency of HCV genotypes in different geographical regions

Frequency of HCV genotypes were examined by individual Provinces shown in table 2. Among the genotypes determined from 2165 Punjabi patients, 7.94% (*n *= 172) belonged to genotype 1a, 2.72% (*n *= 59) genotype 1b, 0.18% (*n *= 4) genotype 1c, 6.46% (*n *= 140) genotype 2a, 0.69% (*n *= 18) genotype 2b, 0.09% (*n *= 2) genotype 2c, 52.10% (*n *= 1128) genotype 3a, 16.53% (*n *= 358) genotype 3b, 0.64% (*n *= 14) genotype 3c, 1.15% (*n *= 25) genotype 4, 0.23% (*n *= 5) genotype 5a, 0.05% (*n *= 1) genotype 6a, and 4.34% (*n *= 101) were dual genotypes. From NWFP 823 cases were grouped after genotyping as follows: subtype 1a in 54 (6.56%); 1b in 21 (2.55%); 2a in 63 (7.65%); 2b in 7 (0.85%); 2c in 1 (0.12%); 3a in 397 (48.23%); 3b in 157 (19.07%); 3c in 7 (0.85%), genotype 4 in 19 (2.30%), 5a in 1 patient (0.12%); 6a in 3 (0.36%); mixed in 42 (5.10%) and untypeable in 51 (6.19%) patients. In Sindh genotype 3a was predominant in being found in 95 (39.74%) of samples followed by 3b (26.35%), 2a (9.62%), 1a (9.2%), 1b (3.76%), 3c (1.25%) and genotype 4 (0.41%). Twelve (5.02%) mixed genotypes were also isolated from this area and 10 (4.18%) samples were found untypeable. Among patients from Balochistan, genotypes 1a (25.8%) was the most prevalent followed by 2a (20.96%), 3a (19.35%), and 3b (11.29%); 1b (9.67%); 2b (10.8%); genotype 4 (4.03%); 1c (0.80%) and 3c (0.80%). Six (4.83%) of the isolates were found with dual infection and 2 (1.61%) were untypeable.

**Table 2:** Prevalence of HCV genotypes in different geographical regions of Pakistan (N = 3351).

***Genotype***	***Subtype***	***Isolated from Punjab***	***Isolated from NWFP***	***Isolated from Sindh***	***Isolated from Balochistan***	***P value <***
**1**	1a	172 (7.94%)	54 (6.56%)	22 (9.20%)	32 (25.80%)	0.01
	1b	59 (2.72%)	21 (2.55%)	9 (3.76%)	12 (9.67%)	0.05
	1c	04 (0.18%)	0	0	01 (0.80%)	-
**2**	2a	140 (6.46%)	63 (7.65%)	23 (9.62%)	26 (20.96%)	0.01
	2b	18 (0.69%)	7 (0.85%)	1 (0.41%)	1 (0.80%)	-
	2c	02 (0.09%)	01 (0.12%)	0	0	-
**3**	3a	1128 (52.10%)	397 (48.23%)	95 (39.74%)	24 (19.35%)	0.01
	3b	358 (16.53%)	157 (19.07%)	63 26.35%)	14 11.29%)	0.01
	3c	14 (0.64%)	07 (0.85%)	03 (1.25%)	01 (0.80%)	-
**4**	4	25 (1.15%)	19 (2.30%)	1 (0.41%)	5 (4.03%)	NS
**5**	5a	5 (0.23%)	1 (0.12%)	0	0	-
**6**	6a	1 (0.05%)	3 (0.36%)	0	0	-
	Mixed^α^	101 (4.34%)	42 (5.10%)	12 (5.02%)	6 (4.83%)	NS
	Undetermined^β^	138 (6.37%)	51 (6.19%)	10 (4.18%)	02 (1.61%)	NS
						
**Total**		**2165**	**823**	**239**	**124**	**3351**

^α^Mixed, two genotypes in one patients such as 3a+1a, 3a+3b etc.

^β^Undetermined, as the genotype assay was unable to assign type and these may be new subtypes.

### Prevalence of HCV mixed-genotype infections

Table 3 shows the prevalence of HCV mixed-genotype infections determined by this system in different populations across Pakistan. Total 161 HCV isolates were found having two genotypes. Of these 101 belonged to Punjab region, 42 to N.W.F.P., 12 to Sindh region and 6 to Balochistan region. More than half of the total HCV samples with mixed infection were infected with HCV genotypes 3a and 3b (49.68%) followed by 3a + 1a (11.18%). The overall rate of HCV mixed-genotype infections in patients with chronic hepatitis C was 6.98%, which was significantly lower than that in the thalassaemic patients (P < 0.001).

**Table 3:** Prevalence of HCV mixed genotypes in Pakistan (n = 161).

***HCV isolate with Mixed genotypes***	***Isolated from Punjab***	***Isolated from N.W.F.P***	***Isolated from Sindh***	***Isolated from Balochistan***	***N***
3a + 3b	56	16	4	4	80
3a + 1a	10	5	2	1	18
3a + 1b	5	4	0	1	10
3a +2a	5	2	2	0	09
1a + 1b	5	3	1	0	09
1a + 3b	4	2	1	0	07
3a + 1c	3	3	1	0	07
2a + 1b	4	1	0	0	05
3b + 2a	3	1	0	0	04
3a + 2b	2	1	0	0	03
1a + 4	0	2	1	0	03
3a + 4	1	1	0	0	02
2a + 1a	1	1	0	0	02
3a + 6a	1	0	0	0	01
					
**Total**	**101**	**42**	**12**	**6**	**161**

N.W.F.P, North West Frontier Province; HCV, hepatitis C virus

### Possible risk factors for infection transmission in various genotypes

Various possible risk factors responsible for infection transmission with each HCV genotypes are given in table 4. Over all the probable modes of transmission observed were: 61.45% due to multiple use of needles/syringes; 10.62% due to major/minor surgery/dental procedures; 4.26% due to blood transfusion and blood products; 3.90% due to sharing razors during shaving or circumcision by barbers, piercing instruments, nail clipers, tooth brushes/Siwaks; in less than 1% due to needle stick, from infected mother to baby and sexual transmission. For 20.35% subjects the mode of transmission was unclear as no possible routes were observed at the time of survey and they were still sporadic.

**Table 4:** Possible route of transmission of various HCV genotypes (N = 3351).

***HCV***	***Possible Mode of Transmission***				
***HCV Subtype(N)***	**Multiple use of needles/syringes (%)**	**Major/minor surgery, dentil procedures (%)**	**Blood and blood products (%)**	**Sharing razors, piercing instruments, Nail clippers, tooth brushes (%)**	**Sporadic (%)**

**1a **(280)	30 (10.71)	101 (36.07)	6 (2.14)	2 (0.71)	141 (50.35)
**1b **(101)	11 (10.89)	38 (37.62)	2 (1.98)	6 (5.94)	44 (43.56)
**1c **(05)	-	-	1 (20)	1 (20)	3 (60)
**2a **(252)	43 (17.06)	18 (7.14)	09 (3.57)	51 (20.23)	131 (51.98)
**2b **(27)	2 (7.4)	2 (7.4)	1 (3.7)	16 (59.25)	6 (22.22)
**2c **(03)	-	1 (33.33)	-	2 (66.66)	-
**3a **(1644)	1421 (86.43)	88 (5.35)	13 (0.79)	12 (0.72)	110 (6.69)
**3b **(592)	427 (72.12)	43 (7.26)	5 (0.84)	2 (0.33)	115 (19.42)
**3c **(25)	3 (12)	5 (20)	3 (12)	9 (36)	5 (20)
**4 **(50)	11 (22)	19 (38)	13 (26)	1 (2)	6 (12)
**5a **(6)	5 (83.33)	1 (16.66)	-	-	-
**6a **(4)	4 (100)	-	-	-	-
**Mixed **(161)	31 (19.25)	9 (5.59)	85 (52.79)	25 (15.52)	11 (6.83)
**Undetermined **(201)	51 (25.37)	31 (15.42)	5 (2.48)	4 (1.99)	110 (54.72)
					
**Total**	**2039 (61.45)**	**356 (10.62)**	**143 (4.26)**	**131 (3.90)**	**682 (20.35)**

^α^Mixed, two genotypes in one patients such as 3a+1a, 3a+3b etc.

^β^Undetermined, as the genotype assay was unable to assign type and these may be new subtypes.

## Discussion

Data so obtained from different parts of the world have focused on the increasing interest of HCV genotyping by mass screening as it is useful for the solution of epidemiological questions and development of vaccines against HCV. Furthermore it has been shown to be beneficial to facilitate therapeutic decisions and strategies [25-27]. It has been demonstrated that the severity of the disease, its progression and response to therapy may vary according to the genotype [10,28].

Substantial regional differences appear to exist in the distribution of HCV genotypes. Thus knowledge on the distribution of various genotypes in our country is essential for its prognostic implications in chronic hepatitis C infection. In the present study the frequency of various genotypes of HCV present in Pakistan and various risks for possible transmission were observed. All serum samples tested were HCV-RNA positive by PCR and could thus be genotyped. However, genotype-specific PCR products were seen in about 94% serum samples by this molecular biology-based system that is an excellent percent of the sample size. The data of the present study correspond well to previous studies, in which genotypes or serotypes were determined from other regions of the world [29-31]. The presence of various HCV genotypes such as 1a, 1b, 2a, 3a, and 3b reported in this study have also been reported in the earlier studies with predominated genotype 3 in Pakistan [21,22]. Though these studies were done with lower sample sizes, the reported genotypes frequencies are the same as observed in the present study. Additionally six more genotypes such as 1c, 2c, 3c, 4, 5a and 6a were isolated for the first time ever from this country in the present study. The frequency of genotype distribution in the present study also seems to be quite similar to that reported from other South Asian countries such as Nepal [17] and India [32,33] where genotype 3 was found as the predominant genotype but different from those in Japan [34], Thailand [35], Vietnam, USA and Western Europe [36] where genotype 1 is the most prevalent HCV genotype.

When the results of the present study on HCV genotypes were compared to the genotyping results obtained in other studies from Pakistan [21,22], it was observed that the frequency of genotype 1 is increasing in this country without any increase in the frequency of genotype 3. It seems that in coming 15–20 years the current most prevalent genotype 3a will be replaced by less common genotype 1 (a or b). If this actually happens here in Pakistan, it will complicate more the present situation of HCV that is a big health problem in this country. Recently replacement of one HCV genotype (type 1b) by another (type 2) has been reported from Venezuela that took only 10 years time for this displacement [36]. We also suggest confirming this observation by conducting other studies on HCV genotypes. Another interesting finding of the study is the isolation of genotype 4 for the first time from Pakistan although this genotype is believed to be absent from Pakistan [22]. HCV genotype 4 is prevalent in the Middle East and Central Africa; however, our patients did not have a history of visit to these areas of the world. Genotype 4 has been reported to be frequently associated with cirrhosis and a poor response to interferon [37,38]. Like Pakistan, HCV genotype 4 is also rare in the United Sates and there are few published data regarding response to therapy in patients with HCV genotype 4 infections in Pakistan and United States [39]. None of the genotype 4 having patient has history of visit to Middle-East.

In order to discern if there are any regional differences in HCV genotype distribution in Pakistan, the distribution for this population was examine by individual Provinces. No regional difference with respect to HCV genotype distribution was seen in the Provinces of Punjab, N.W.F.P and Sindh where the most prevalent genotype is 3a followed by 3b. But a clear difference was observed in the Province of Balochistan where the predominant genotype found was 1a followed by 2a and 3a. Balochistan shares a long boarder with Iran in the west where genotype 1 is the predominant genotype in the region [40]. It is quite possible that genotype 1 may enter to Pakistan from Iran through local persons who cross boarder for jobs and trade. HCV genotypes distribution described in this study does not present modification by gender, and the distribution of HCV genotypes was similar both in male and female HCV patients with no significant difference seen for various genotypes in male and female patients (P < 0.05). The results of the present study indicate that HCV genotype distribution varies with age. It has already been reported that HCV genotype distribution varies with age in both male and female patients [41]. Subtypes 1a and 1b were found most prevalent among older patients, whereas subtype 2a/2b and 3a/3b were mainly found among younger ones.

Some studies suggest that different types of HCV may be associated with different transmission routes. In the present study majority of the patients seem to have more than one route of transmission however we took the most probable one for data analysis. In Pakistan genotype 3a is the most prevalent genotype and more than 86% patients with genotype 3a received multiple injections. It is quite possible that this type was spread in Pakistan by doctors, vaccination teams and other medical persons using non-disposable syringe before 1990 when one syringe was used for injections to all attended patients. This type of practice is still there in the rural areas of the country. This observation is supported by a study where subtype 3a appeared to be prevalent among injection drug users and it is believed that it was introduced into North America and the United Kingdom with the widespread use of heroin in the 1960s [42]. Further, it has also been reported that HCV genotype 3a is particularly prevalent in intravenous drug abusers in Europe and the United States [43]. For genotype 1a and 1b, the probable causes of transmission observed in the present study were major/minor surgeries and dental procedures. This is in agreement with a study from Hungry where majority of the cases with genotype 1 has a history of hospitalization for major/minor surgery, dental procedures and shaving by barbers. In contrast in one study, 95% of all patients with genotype 1 were infected with subtype 1b and majority of those patients had received transfusions of blood and/or blood products [30]. Genotype 2a was more frequently seen (>50%) in case with history of no apparent causes of transmission. Mode of transmission was not clear for more than half of patients with untypable genotypes. Type 4 is uncommon in this country and its route of transmissions were surgeries (38%) followed by reuse of syringes (22%). As expected, the prevalence of HCV mixed-genotype infections was high (52.79%) in thalassaemic patients who had received multiple blood transfusions.

It appears that more than 70% of the cases were acquired in hospitals or other medical facilities because a large number of subjects (61.45%) received therapeutic injections and surgeries (10.62%) from the clinics of local general practitioners in both government and private hospitals. It has already been reported that Pakistan has one of the highest frequencies of injections in the world and the average number of injections per person per year is more than 9 injections (44–45). Further been reported in the same studies that 49% of patients receive injections at their first outpatient visit where in addition to the unnecessary use of injections, safe injection practices are not followed. Furthermore, previous vaccination have also been alleged to ignore safe injection practices that was largely received at the public health-care facilities including the use of used syringes [44,45]. As shown in the present study and also reported in previous other studies [44,45] subjects who received more injections were more likely to be infected with HCV and these non-sterile syringes or needles may be the source of HCV infections. According to a survey conducted by Ministry of Health, in Pakistan more than 72% therapeutic injections and 50% immunization injections in public health-care facilities are unsafe and potentially dangerous [46]. In these studies it is further shown that the use of multiple-dose vials is common in many government and private sector hospitals of Pakistan that is the major risk factor for the transmission of HCV infection [44-46] especially genotype 3a.

## Conclusion

The predominant HCV genotype in Pakistan is type 3a followed by 3b and 1a. Six HCV genotypes such as 1c, 2c and 3c, 4, 5a and 6a were isolated for the first time from Pakistan during the study. The frequency of genotype 1 was observed to be increasing in this country without any increase in the frequency of genotype 3 that may be very dangerous in coming 15–20 years. Regional difference in genotypes was observed only in Balochistan province of Pakistan. More than 70% of the cases in Pakistan are hospitals acquired.

## Competing interests

The author declares that they have no competing interests.

## Authors' contributions

SR conceived of the study, participated in its design and coordination and gave a critical view of manuscript writing. MI collected epidemiological data and analyzed the data statistically. MI carried out the molecular genotyping assays. All the authors have read and approved the final manuscript.

## Pre-publication history

The pre-publication history for this paper can be accessed here:


